# An Illustrated Dictionary of Medicine, Biology, and Allied Sciences

**Published:** 1894-07

**Authors:** 


AN ILLUSTRATED DICTIONARY OF MEDICINE, BIOLOGY, AND ALLIED SCIENCES. By George M. Gould, A. M., M. D. Based upon recent scientific literature. Philadelphia: P. Blakiston, Son & Co., 1012 Walnut Street. 1894.

This work is a large quarto of over sixteen hundred pages, containing the latest information on medical subjects possible to obtain. The author, some three years ago, got out a very good dictionary of octavo size, which was, in its way, a handy volume to have on the rack for ready reference.

In the present volume Dr. Gould has abandoned his first effort, and starting anew, has created a handsome work many times larger than the one of which we have just spoken.

The author says in the preface, "I have conscientiously endeavored to give the greatest possible amount of lexicographic and encyclopædic knowledge within the limits of a single volume, condensed as much as is consistent with clearness, and so arranged as to furnish the student and worker with concise, accurate, and useful definitions." It therefore includes many thousands of new words and terms that have been introduced into medicine during the last few years: To give the most compact *résumé* of the works of older and authoritative lexicographers: To include all the more commonly-used terms of biology—a thing highly desirable for three reasons: first, because of the modern recognition of the great truth that biological science is the foundation of genuine and progressive medical science; secondly, because the best schools of medicine are more and more urging or making obligatory the preliminary biological course of study; and third, because no satisfactory lexicon of biology exists in English.

In addition to the usual pronunciation, derivation, and definition of words, there is also given their logical relations, bearings, and practical importance. There are many tables of the most valuable character, and a marvelous number of fine illustrations.

There is such a number of fine dictionaries of various kinds before the intellectual public that one would think there were too many to survive. Yet when they are individually examined each has excellent qualities peculiarly its own, which give it a place of which it cannot be robbed.

It is so with the dictionary under review. It has characteristics, some of which have been enumerated, which make it desirable and, indeed, indispensable to every enlightened practitioner.

The writer of this notice confesses to a strong admiration for dictionaries; for he appreciates the vast field of diverse information included within the limits of one book; he realizes and admires the comprehensiveness of mind and diversity of knowledge that inspires the production of such a work; the immense and persevering industry required to accomplish the purpose; and the commercial courage required to put capital into the manufacture of such a costly magazine of learning.

With pleasure then he turns over the handsomely printed pages, and records his impressions in this notice for the edification of the readers of this journal.

The homœopathic physician will, of course, naturally turn to the definition of Homœopathy. While not as satisfactory as might be desired, nevertheless, it is free from the offensive and perverse misrepresentations which have characterized the older medical dictionaries, and, therefore, it will pass without exciting active annoyance.

This is the more gratifying as well as surprising, as the author has made himself very conspicuous lately on account of his vicious attacks on the homœopathic school. These attacks have created a certain amount of antagonism in our ranks toward any production of Dr. Gould's pen, however meritorious. This antagonism, however, is most bitter among those who are not strict homœopaths, but admit a certain amount of old-school therapeutic practice into their own reasonings and remedial measures under the guise of *liberal* Homœopathy. The strict homœopaths have not felt hurt by these attacks and have in most instances been quite unconscious of them. These will not have their judgment clouded as to the merits of the book, nor should there be any resentment toward the book because of its author in the minds of the others—the *liberals*—since it is not the author who is under review, but a book, and that a book a dictionary—a valuable miniature encyclopædia.

